# Essential Nutrient and Trace Element Foliar Resorption of Two Co-Existing *Nothofagus* Species Grown Under Different Environmental Conditions in Southern Patagonia

**DOI:** 10.3389/fpls.2019.01542

**Published:** 2019-11-27

**Authors:** Héctor A. Bahamonde, Victoria Fernández, Javier Gyenge, Francisco Mattenet, Pablo L. Peri

**Affiliations:** ^1^Department of Forestry Research, Instituto Nacional de Tecnología Agropecuaria (INTA), Santa Cruz, Argentina; ^2^Department of Natural Resources, Universidad Nacional de la Patagonia Austral (UNPA), Río Gallegos, Argentina; ^3^Forest Genetics and Ecophysiology Research Group, School of Forest Engineering, Technical University of Madrid, Madrid, Spain; ^4^Department of Ecophysiology Research, Consejo Nacional de Investigaciones Científicas y Técnicas (CONICET), Buenos Aires, Argentina; ^5^AER Tandil, EEA Balcarce INTA, Tandil, Argentina

**Keywords:** mineral nutrition, native forest, Patagonian *Nothofagus*, deciduous trees, nutrient cycling

## Abstract

Nutrient resorption is crucial for mineral element conservation and efficiency of forest species, but knowledge on its significance and the mechanisms involved is still limited for most species and habitats. Focusing on the harsh conditions for plant growth and survival of southern Patagonia, a field study for comparing the rate of foliar resorption of macro-, micro-nutrients, and trace elements in coexisting *Nothofagus pumilio* and *Nothofagus antarctica* forests was performed. Forests located in three contrasting productivity sites (with different soil and climatic conditions) were selected, and mature, functional versus senescent leaves of both species were collected at two different dates of the growing season. Macro- (N, P, Ca, K, S, and Mg), micronutrients (B, Cu, Fe, Mn, Zn, and Ni), and trace elements (Al, Li, Pb, Rb, Sr, Ti, and Tl) were determined in foliar tissues. The mineral element concentrations of mature and senescent leaves were used for calculating the nutrient resorption efficiency (NuR). In general, and making an average of all sites and species, macro-nutrient resorption showed a decreasing trend for N > S = K > P > Mg, being Ca the only macro-nutrient with negative values (i.e., no resorption). Resorption of the majority of the elements did not vary between species in any of the evaluated sites. Variation across sites in nutrient resorption efficiency for most macronutrients, some micronutrients, and trace elements was observed for *N. antarctica*, whereas *N. pumilio* had a similar NuR for all experimental sites. On the other hand, regardless of the site or the species, some elements were not resorbed (e.g., B, Cu, Fe, Mn, Al, and Ti). It is concluded that both *Nothofagus* species performed similarly concerning their nutrient conservation strategy, when coexisting in the same mixed forest. However, no evidence was gained for an increased rate of foliar NuR in association with the sites subjected to more limiting soil and climatic conditions for plant growth.

## Introduction

Net primary productivity in temperate forests is strongly related to internal nutrient cycling (Schlesinger, 1991). The “movement” of nutrients from leaves prior to abscission toward other tissues and/or storage organs has been indicated as a key component of nutrient conservation in forests of the whole world (Ares and Gleason, 2007; Freschet et al., 2010), being the term “nutrient resorption” (NuR) widely accepted nowadays (Killingbeck, 1996). This strategy allows plants to use the resorbed nutrients for new growth and/or storage in vegetative tissues for further growth during the next seasons (Van Heerwaarden et al., 2003), hence minimizing their dependence on the available soil nutrient pool. Several studies on NuR in trees focused on macronutrients [mainly nitrogen (N) and phosphorus (P)] as main elements limiting growth in forest ecosystems (Hagen-Thorn et al., 2006). At a global scale, terrestrial plants can resorb more than 60% of N and P (Vergutz et al., 2012). The resorption of other macronutrients [e.g., calcium (Ca), potassium (K), and magnesium (Mg)] and micronutrients [e.g., copper (Cu) or zinc(Zn)] has been little explored to date (Vergutz et al., 2012) despite their important role in plant metabolism and ecosystem functioning (Vitousek and Howarth, 1991; Liu et al., 2014). On the other hand, some studies suggested that a higher NuR efficiency is more common in plants grown on low nutrient soils (Pugnaire and Chapin, 1993; Wang et al., 2018) or that it may increase with soil nutrient availability (Fisher and Binkley, 2000). Additionally, other researchers observed no relationship between NuR and soil nutrient availability (Aerts, 1996; Diehl et al., 2003). Nevertheless, there is evidence that the resorption process is affected by environmental conditions, such as increased temperatures or drought (Lawrence and Melgar, 2018). Some authors reported, for example, a strong influence of latitude, mean annual precipitation, and temperature on N and P resorption (Yuan and Chen 2009; Zhao et al., 2017).

Nowadays, 17 elements from the periodic table are recognized as essential macro- and micro-nutrients for plants (Marschner, 2012). There are also other elements such as aluminum (Al; Pilon-Smits et al., 2009) or titanium (Ti; Lyu et al., 2017) which at low concentrations are considered beneficial for some species. Regarding other trace elements, some are considered beneficial at low concentrations while at high concentrations are regarded as heavy metals and toxic for plants. For instance, supply of low amounts of lithium (Li) to some species have been found to improve plant growth, but Li toxicity has been reported in many areas of the world (Shahzad et al., 2016). The detrimental effects of plant absorption of heavy metals such as lead (Pb; Pourrut et al., 2011), thallium (Tl; Mazur et al., 2016), or strontium (Sr; Sasmaz and Sasmaz, 2017) have been shown in several studies. Rubidium (Rb) has often been used as marker for K in plant nutrition studies, but plants can apparently discriminate between K and Rb and K shortage facilitates the uptake of closely related Rb (Nyholm and Tyler, 2000). All such essential, beneficial, and trace elements may be available in soils and taken up by plants, but there is limited information about the occurrence and potential rate of resorption in forest ecosystems.

Native forests in southern Argentinean Patagonia (Santa Cruz and Tierra del Fuego provinces; 46° to 55° latitude) cover approximately 1.2 million ha, where southern beeches [i.e., *Nothofagus pumilio* (lenga) and *N. antarctica* (ñire)] represent more than 95% of the total forested area (Peri et al., 2019). In general, both deciduous species grow in different sites under contrasting environmental conditions. *Nothofagus pumilio* forests are mostly found as pure stands (despite in Tierra del Fuego Island they may be associated with evergreen *N. betuloides* in transitional zones) in well-developed and adequately-drained soils. By contrast, pure *N. antarctica* (a more plastic species) forests can be found in sites with an array of limiting factors for plant growth, such as rocky and poorly-drained soils or xeric environments in the limit with the Patagonian steppe (Frangi et al., 2004). The growth rate differences between *Nothofagus* species, growth being higher in *N. pumilio* than *N. antarctica*, determine small transitional areas where both species may co-exist. Working under controlled conditions, Peri et al. (2009) determined higher leaf net photosynthesis and stomatal conductance rates for well-watered *N. pumilio* versus *N. antarctica* seedlings. However, when water became a stress factor (both drought and flooding) *N. antarctica* seedlings had a better performance. Similarly, among the different *Nothofagus* species analyzed by Dettmann et al. (2013), *N. antarctica* was found to be the one having the smallest xylem vessel diameters which may contribute to reduce the loss of hydraulic conductance caused by freeze-induced embolism. These traits may partially explain the lower growth rate of *N. antarctica* in comparison to *N. pumilio* and their distribution under natural conditions, but knowledge on the ecophysiology of such species is still scarce and fragmentary. Furthermore, these different features of both species may also be related to their capacity to absorb nutrients from the soil and to resorb them prior to leaf abscission. Nutritional aspects of such species have been little explored and chiefly focused on few macronutrients, but increased nutrient concentrations (N, K) and higher N, P, and K resorption rates for *N. pumilio* versus *N. antarctica* have been determined in Andean forest of northern Patagonia (Diehl et al., 2003).

In an altitudinal gradient study carried out in *N. pumilio* forests of Tierra el Fuego Island, where the productivity of the stands decreased at higher altitudes (Barrera et al., 2000), Frangi et al. (2005) suggested that the retranslocation of mobile nutrients may increase with elevation. According to the results obtained by Peri et al. (2006, 2008) N, P, K, and sulfur (S) concentrations in *N. antarctica* green leaves were higher in stands growing in better sites (with more favorable soil and/or environmental conditions) which led to higher aboveground productivities (represented by an average height of mature trees of 8 m and above, compared to marginal sites with mean tree heights below 5.3 m). Similarly, Bahamonde et al. (2018a) observed that higher *N. antarctica* forest stand productivities in southern Patagonia were mainly related to lower altitudes, reduced differences between maximal and minimal daily temperatures, and increased soil depths.

Concerning the NuR efficiency of *Nothofagus* species in Patagonian forests, some investigations indicated differences between species grown under similar environmental conditions, in addition to effects of climatic conditions on the NuR of the same species. For example, Hevia et al. (1999) measured different values of N and P resorption between evergreen *N. dombeyi* and deciduous *N. pumilio* and *N. obliqua*, but they also found variable resorption efficiencies between the two deciduous *Nothofagus* species investigated. Similarly, Diehl et al. (2003) reported a high resorption variability within species which may be associated with different environmental conditions (mainly climatic factors). Fajardo and Piper (2015) also observed a high variability in NuR of evergreen *N. betuloides* and *N. dombeyi* grown in six contrasting experimental sites, with precipitation ranging from 600 to 3000 mm per year. However, the NuR of a particular species under the prevailing climatic conditions in each experimental plot were not specified in this study.

In light of the state of knowledge of NuR of South-American *Nothofagus* species, the aim of this study was to compare the rate of foliar macronutrient, micronutrient, and trace element resorption in coexisting *N. pumilio* and *N. antarctica* forests located in contrasting productivity sites of southern Patagonia. The study is focused on foliar NuR, because previous investigations showed that leaves approximately represent 70% of the mineral element return from above-ground litter in *Nothofagus* spp. forests of southern Patagonia (Frangi et al., 2005; Bahamonde et al., 2015). In addition to essential elements, the resorption of some trace elements which may be either considered toxic or beneficial for plants was also assessed for the first time in Patagonian forests. Subsequently, the following hypotheses were tested: (i) aware of the higher growth potential of *N. pumilio* versus *N. antarctica*, it is assumed that *N. pumilio* will be more NuR efficient than *N. antarctica* trees when grown under similar soil conditions, and (ii) for both species, NuR rates will be higher in stands growing under harsher climatic conditions (i.e., higher altitude, lower temperature, and reduced rainfall) to compensate for their lower growth rate.

## Material and Methods

### Study Sites

The three study sites belong to the PEBANPA network (Peri et al., 2016) and correspond to an environmental gradient, where contrasting altitude, mean annual precipitation, and mean annual temperature led to different dominant, mature, tree heights (as a proxy of stand productivity). This implies that sites with higher dominant trees are located at lower altitude and are exposed to higher temperature and increased precipitation regimes (**Table 1**). Other bioclimatic parameters also differ between experimental sites. For instance, the mean temperatures during the wettest trimester of the year are higher in the most productive site and are lower in less productive locations. In addition, compared to the other experimental zones, temperatures are more homogeneous along the year (lower differences between monthly maximal and minimal temperatures) in the area where the highest trees are located (data not shown).

**Table 1:** Main characteristics of *Nothofagus* forests located in three different sites of southern Patagonia.

Site quality	Geographical coordinates	Altitude (m a.s.l.)	MAP (mm y^−1^)	MAT (°C)	DH (m)	
					**Na**	**Np**
HPS	50°32’55”‘ S—72°48’59’’ W	233	610	7.2	12.3 ± 1.1a	18.5 ± 1.8a
MPS	51°13`21” S—72°16`52” W	300	450	5.9	10.2 ± 1.3b	15.2 ± 1.1b
LPS	51°33’07’’ S—72°07’26’’ W	550	440	5.5	7.2 ± 1.0c	12.6 ± 1.0c

MAP, mean annual precipitation; MAT, mean annual temperature; DH, dominant height of mature trees; Na, Nothofagus antarctica; Np, Nothofagus pumilio.

HPS, highly productivity site; MPS, medium productivity site; LPS, low productivity site.

For DH Different letters for a same species indicate significant differences (P < 0.05) between sites.

In each experimental plot both species grew together as mixed stands. Altitude and climatic variables for each site were obtained from Farr et al. (2007) and the World Clim data set (www.worldclim.org, Hijmans et al., 2005), respectively. At each site the height of six dominant mature trees was measured. Based on the mean height of dominant trees (DH) and previous investigations, we will subsequently refer to site productivity as high (HPS), medium (MPS) and low (LPS). The use of DH as a proxy of productivity is based on the investigations by Pastur et al. (2000) and Ivancich et al. (2011) for *N. pumilio* and *N. antarctica*, respectively. These authors measured tree height and area, also estimating the wood volume of *Nothofagus* forests growing under a vast array of representative environmental conditions of southern Patagonia. For such purpose, they introduced site index equations and obtained accurate models that related DH with productivity. In addition, the age of mature dominant trees was determined by ring counts, being an average of 151, 149, and 153 years for *N. antarctica* in HPS, MPS, and LPS, respectively. In the case of *N. pumilio*, dominant trees had a mean age of 195, 199, and 200 years in HPS, MPS, and LPS, respectively.

### Leaf and Soil Sampling and Nutrient Analyses

At the end of February 2015 (i.e., the peak of the growing period) for both species (*N. pumilio* and *N. antarctica*) and for the three sites subjected to investigation (**Table 1**), approximately 1000 fully-expanded, mature, green leaves were randomly collected from five trees per experimental plot (200 leaves per tree). Thereafter, in autumn (i.e., late May in the Southern hemisphere), a similar leaf collection process from the same marked trees was carried out for sampling senescent leaves which were already yellow. For both species and dates, leaf tissues were collected from fully sun-exposed branches/leaves at a tree height of 2.0–2.5 m above ground level. Leaf samples were immediately taken to the lab in closed plastic bags and kept at 3°C in the refrigerator. Leaf tissues were thoroughly washed in an acidulated solution containing 0.1% detergent (Cif, Argentina) (Álvarez-Fernández et al., 2001; Venturas et al., 2014), and then rinsed with abundant tap and distilled water. Leaves were subsequently oven dried at 70°C during 2 days, weighted, and ground prior to mineral element determination. Nitrogen was measured with an elemental analyzer (TruSpec). The remaining elements were determined by inductively coupled plasma (ICP) analysis (Optima 3000, PerkinElmer) following the UNE-EN ISO/IEC 17025 standards for calibration and testing laboratories (CEBAS-CSIC Analysis Service, Murcia, Spain).

The mineral element concentrations of mature (Nmat) and senescent (Nsen) leaves were used to calculate the NuR efficiency (NRE), according to the following formula (Aerts 1996):

NRE=((Nmat−Nsen)/Nmat) x MLCF) x 100

Where MLCF is the mass loss correction factor. Specifically, it represents the ratio of the dry mass of senescent leaves and the dry mass of green leaves (van Heerwaarden et al., 2003). For each experimental location and species, the MLFC was calculated per sampled tree.

For each sampling date and site, ten fresh leaves of both species were scanned after removing the petioles, using ImageJ software (Rasband, 2004) to calculate their leaf area. Leaves were subsequently dried at 65°C for 3 days and weighted for calculating the specific leaf area (SLA) (Bahamonde et al., 2018b).

Additionally, three composite soil samples (from 5 subsamples) of the first 20 cm were collected from each site corresponding to places where leaves were sampled. This sampling depth was selected aware that in such soils 70% of fine roots have been found with the upper 20 cm of soil (Bahamonde et al., 2016a). Soil pH was determined in saturated soil paste (30 ml deionized water per 100 g soil). Organic matter (OM) content was measured according to the loss of ignition method. Samples were dried to eliminate water, subsequently heated for 2 h at 600°C and the weight loss recorded (Nadal et al., 2004). Soil nutrient concentrations were determined as follows: total N was measured by spectrophotometry; available P by the Olsen method; ammonium acetate-extracted Mg, Ca, K, and sodium (Na) concentrations were determined by atomic absorption spectroscopy (AAS); while the concentrations of diethylene triamine pentaacetic acid (DTPA)-extractable iron (Fe) and manganese (Mn) were also determined by AAS (AQM Laboratories, Spain).

### Data Analysis

Exploratory testings were carried out to verify the compliance with the assumptions of normality, homoscedasticity, and independence of data for each evaluated situation. While the Shapiro–Wilk test was performed to verify the normality of the data, the Levene test was used to verify homoscedasticity. The independence was verified by analyzing residuals from graphs. Specific leaf area and leaves nutrient concentrations were analyzed with ANOVA for repeated measures with species as between subject factor and each measuring date as within subject factor. This analysis was performed because the values are not independent of time. Furthermore, for each species one-way ANOVA analyses were carried out for comparing SLA and nutrient concentrations. Mean values of NuR and minerals soil concentration were compared by two-way ANOVA, with species and sites as main factors. Tukey tests were performed to assess differences between factors when F-values were significant (P < 0.05).

## Results

The specific leaf area (SLA) was assessed for mature (green) and senescent leaves of both *Nothofagus* species and values were within a similar range. However, higher SLA values were recorded for senescent leaves of both species (P < 0.05). No differences between sites were found for any of the two species (P > 0.05) (**Table 2**). Only in the LPS there were differences between species (P < 0.05), being higher in *N. pumilio* mature leaves.

**Table 2:** Specific leaf area (SLA) values ± standard deviation of *N. pumilio* and *N. antarctica* mature and senescent leaves collected from three contrasting sites (HQS, MQS and LQS).

Sites	Species	SLA(m^2^ kg^−1^)	
		**Mature**	**Senescent**
HPS	*N. pumilio*	14.2 ± 2.2b	16.9 ± 1.5a
	*N. antarctica*	14.7 ± 2.4a	14.7 ± 2.7a
Significance		ns	ns
MPS	*N. pumilio*	14.8 ± 1.5b	18.0 ± 1.8a
	*N. antarctica*	12.2 ± 1.3b	16.9 ± 3.0a
Significance		ns	ns
LPS	*N. pumilio*	15.3 ± 2.7b	18.7 ± 3.1a
	*N. antarctica*	12.0 ± 2.9b	16.6 ± 0.6a
Significance		*	ns

HPS, highly productivity site; MPS, medium productivity site; LPS, low productivity site.

Different letters for a same species and site indicate significant differences (P < 0.05) between stage of sampling (mature or senescent).

Significant differences between species for a same stage of sampling and site are given by *: P < 0.05; ns: no significant differences.

### Concentration of Nutrients, and Trace Elements in Leaf Tissues and Soils

Concerning the characteristics of forest soils, only organic matter and N concentrations were different between experimental sites (P < 0.05), both parameters being higher in the LPS (low productivity site, **Table 3**).

**Table 3:** Macro- and micro-nutrients ± standard deviation in soils (0–20 cm) of *N. pumilio* and *N. antarctica* forests from three sites with contrasting environmental conditions. Organic matter (OM) and nitrogen (N) concentrations are expressed as %, while the rest of elements are expressed as mg kg^−^^1^.

Sites	OM	N	P	K	Ca	Mg	Mn	Fe	Na	pH
HPS	13 ± 6ab	0.6 ± 0.2b	59 ± 40	776 ± 429	8219 ± 505	5190 ± 2050	31 ± 25	170 ± 14	523 ± 26	6.3 ± 0.1
MPS	11 ± 2b	0.5 ± 0.1b	18 ± 2	419 ± 29	5610 ± 560	8443 ± 4200	11 ± 1	175 ± 8	542 ± 21	6.4 ± 0.1
LPS	30 ± 11a	1.0 ± 0.2a	64 ± 45	506 ± 59	6468 ± 4514	10523 ± 4100	16 ± 10	195 ± 49	534 ± 63	6.1 ± 0.5

HPS, highly productivity site; MPS, medium productivity site; LPS, low productivity site.

Different letters for the same nutrient indicate significant differences (P < 0.05) between sites.

**Figure 1:**
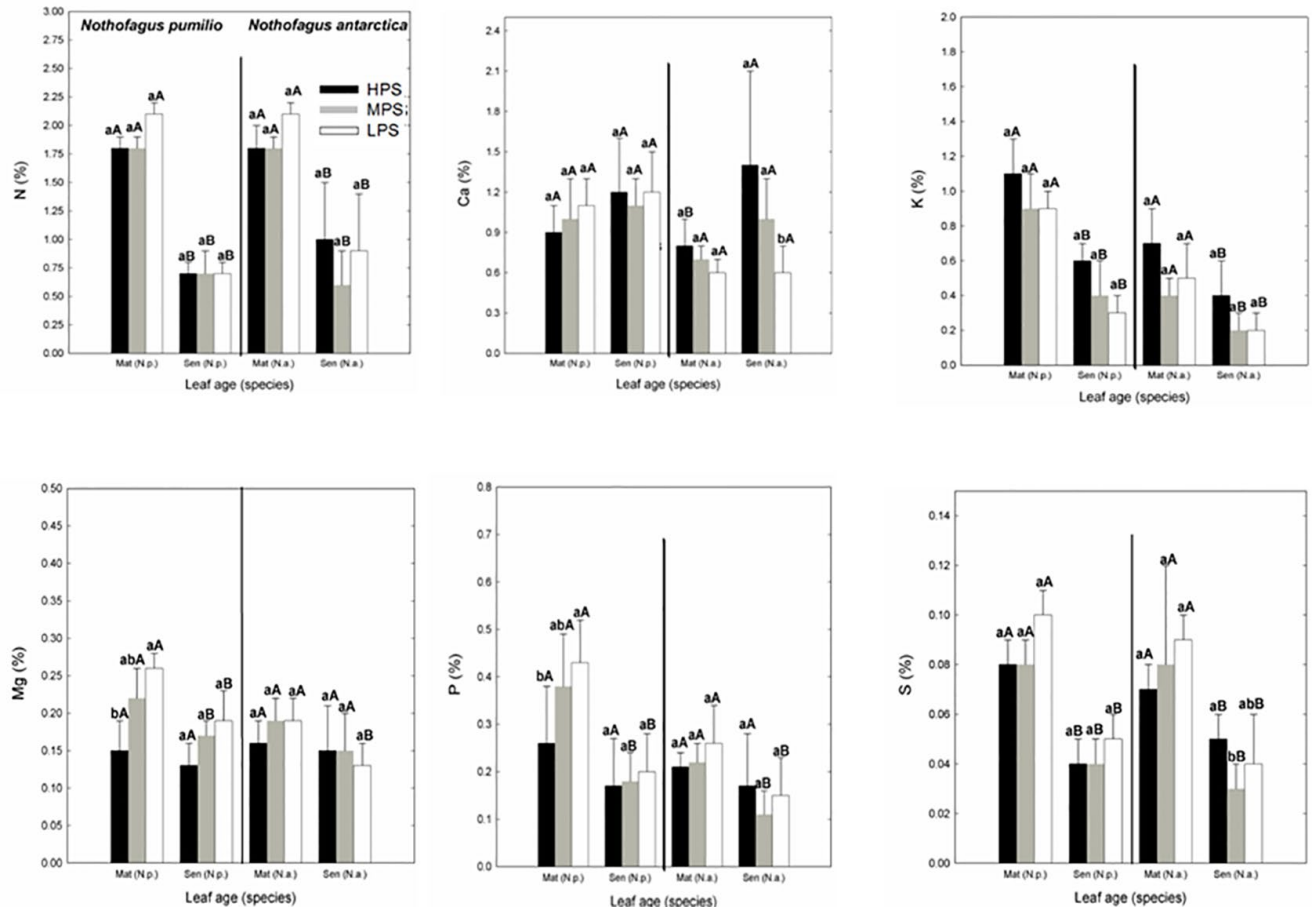
Macronutrient (N, P, Ca, K, Mg, and S) concentrations of *N. pumilio* and *N. antarctica* leaves from three contrasting sites, collected at two developmental stages (mature and senescent). HPS, highly productivity site; MPS, medium productivity site; LPS, low productivity site. N.p., *Nothofagus pumilio*; N.a., *Nothofagus antarctica*; Mat, mature leaves; Sen, senescent leaves. Different lower case letters for the same species and age of leaves (mature or senescent) indicate significant differences (P < 0.05) between sites. Different capital letters for the same species and site indicate significant differences (P < 0.05) between age of leaves (mature or senescent). Bars represent the standard deviation of the mean.

In general, for both species the macronutrient concentrations of leaves collected at the same date did not differ between sites. Only Mg and P concentrations were significantly lower (P < 0.05) in the LPS in *N. pumilio* mature leaves (**Figure 1**). In *N. antarctica*, only Ca and S concentrations were higher (P < 0.05) in the HPS and MPS, respectively in senescent leaves. On the other hand, when comparing between mature and senescent leaves for a particular species and site, most of the macronutrient concentrations were significantly lower (P < 0.05) for senescent leaves (**Figure 1**). The N:P ratio of leaves in all evaluated situations averaged the value of 9 or below (data not shown). There were no differences (P > 0.05) on N:P ratios between sites for the same species and leaf age, or between leaf age for a same species and site.

**Figure 2:**
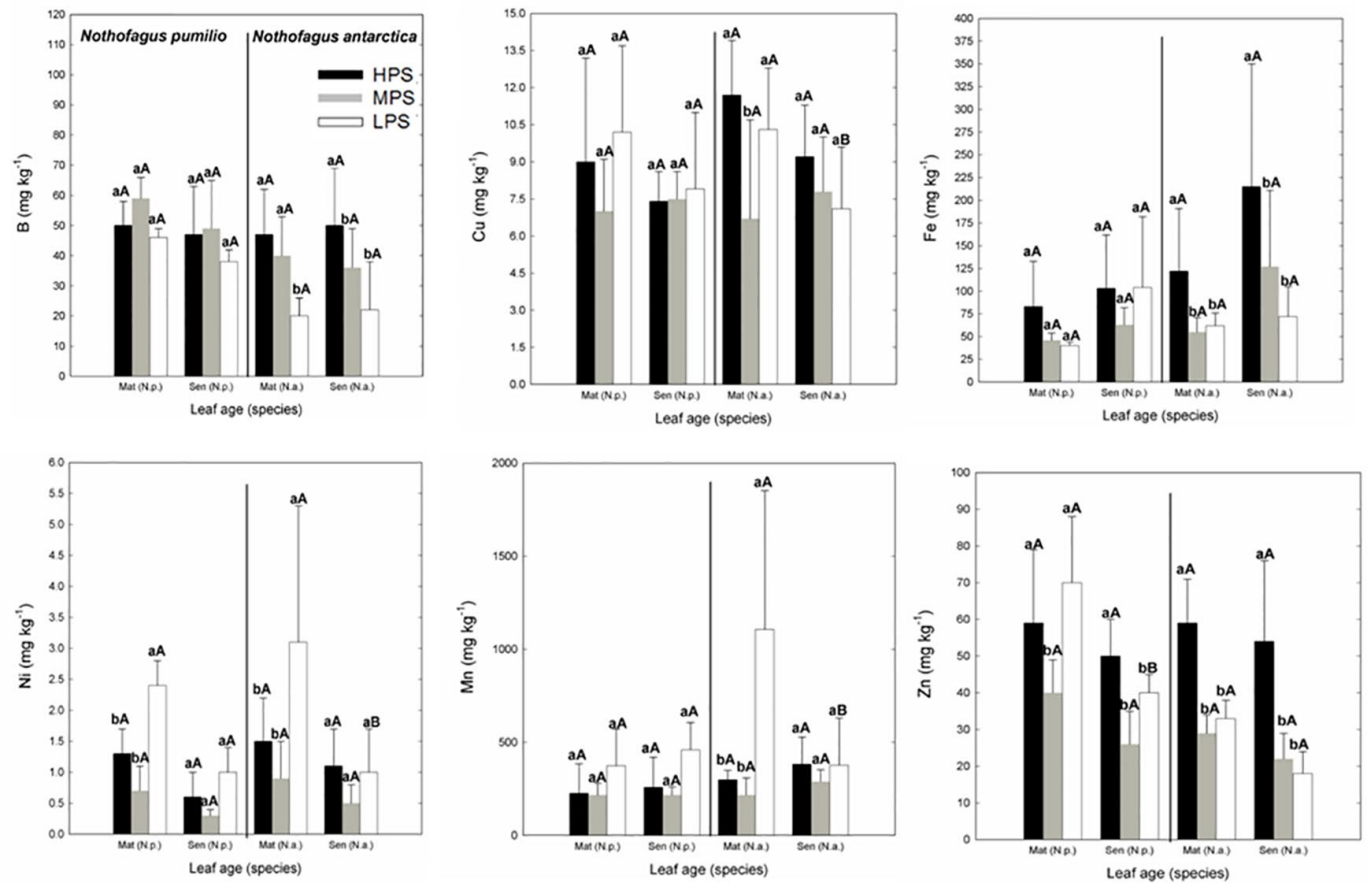
Micronutrient (B, Cu, Fe, Mn, Ni, and Zn) concentrations of *N. pumilio* and *N. antarctica* leaves from three contrasting sites, collected at two developmental stages (mature and senescent). HPS, highly productivity site; MPS, medium productivity site; LPS, low productivity site. N.p., *Nothofagus pumilio*; N.a., *Nothofagus antarctica*; Mat, mature leaves; Sen, senescent leaves. Different lower case letters for the same species and age of leaves (mature or senescent) indicate significant differences (P < 0.05) between sites. Different capital letters for the same species and site indicate significant differences (P < 0.05) between age of leaves (mature or senescent). Bars represent the standard deviation of the mean.

In the case of micronutrients, there were differences between sites (P < 0.05) depending on the particular element (**Figure 2**). For example, Fe and Zn concentrations were generally higher in the highly productivity site (HPS), while increased nickel (Ni) concentrations were recorded for trees growing in LPS. On the other hand, differences between mature and senescent leaves were only found for *N. antarctica* trees growing in the LPS (for Cu, Mn, and Ni), and for *N. pumilio* (for Zn) in the same site, with higher values in mature leaves.

**Figure 3:**
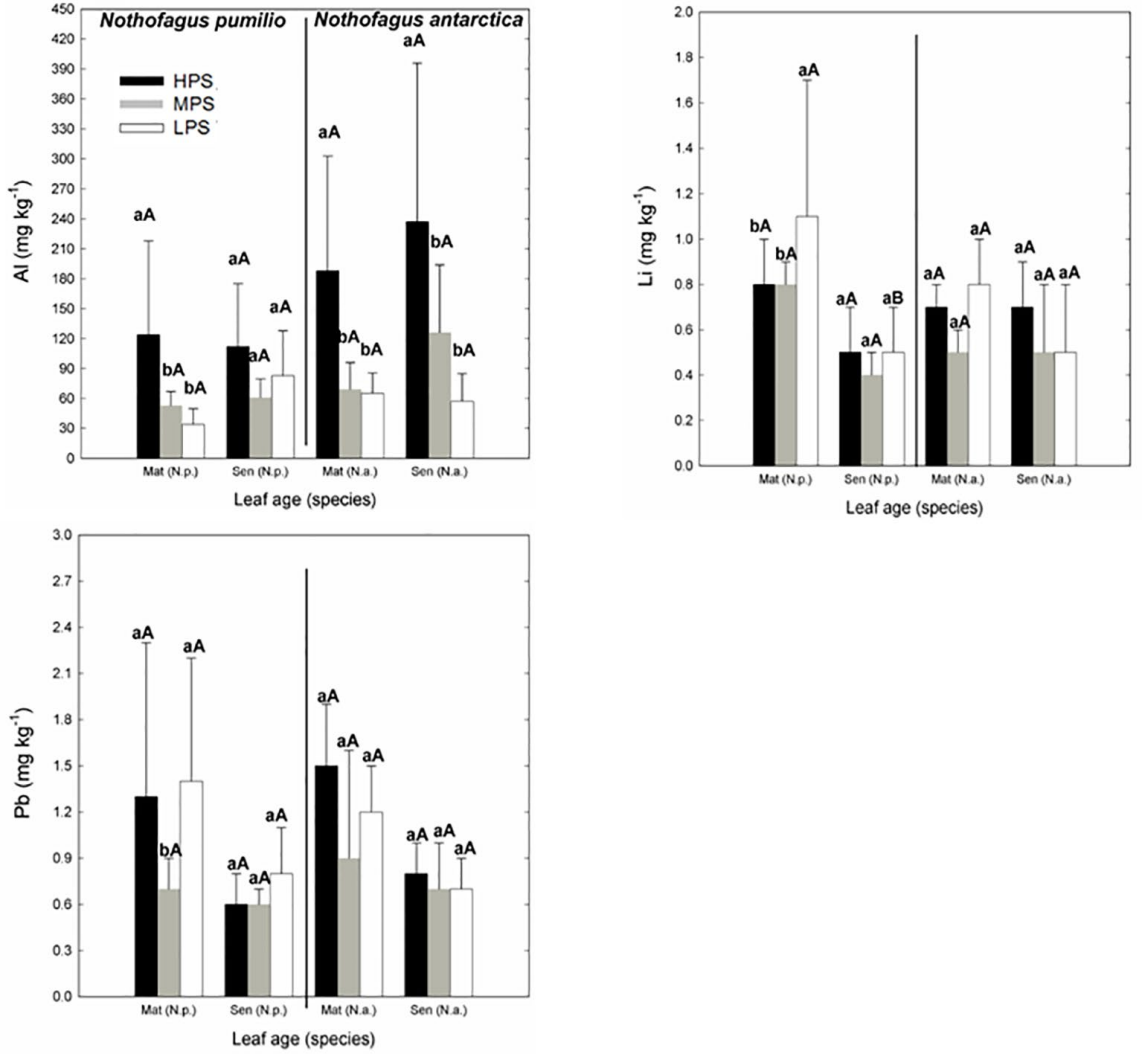
Trace elements (Al, Li, and Pb) concentrations of *N. pumilio* and *N. antarctica* leaves from three contrasting sites and collected at two developmental stages (mature and senescent). HPS, highly productivity site; MPS, medium productivity site; LPS, low productivity site. N.p., *Nothofagus pumilio*; N.a., *Nothofagus antarctica*; Mat, mature leaves; Sen, senescent leaves. Different lower case letters for the same species and age of leaves (mature or senescent) indicate significant differences (P < 0.05) between sites. Different capital letters for the same species and site indicate significant differences (P < 0.05) between age of leaves (mature or senescent). Bars represent the standard deviation of the mean.

**Figure 4:**
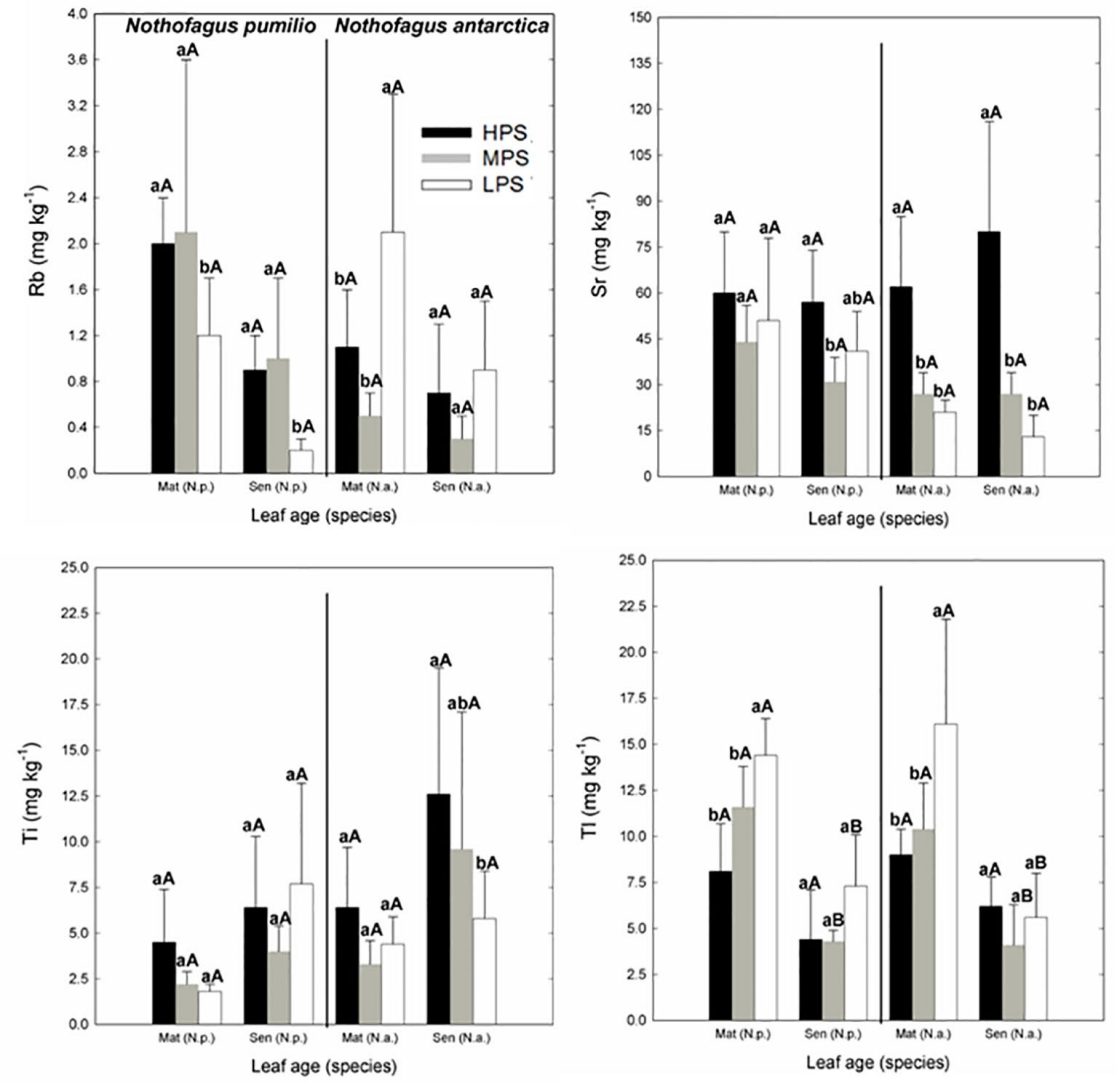
Trace elements (Rb, Sr, Ti, and Tl) concentrations of *N. pumilio* and *N. antarctica* leaves from three contrasting sites and collected at two developmental stages (mature and senescent). HPS, highly productivity site; MPS, medium productivity site; LPS, low productivity site. N.p., *Nothofagus pumilio*; N.a., *Nothofagus antarctica*; Mat, mature leaves; Sen, senescent leaves. Different lower case letters for the same species and age of leaves (mature or senescent) indicate significant differences (P < 0.05) between sites. Different capital letters for the same species and site indicate significant differences (P < 0.05) between age of leaves (mature or senescent). Bars represent the standard deviation of the mean.

Concerning trace elements (**Figures 3** and **4**), for mature leaves of both species Al and Sr concentrations were generally higher (P < 0.05) in the best site (HPS), while higher leaf Tl concentrations were recorded for trees growing in LPS. The only trace elements which varied significantly (P < 0.05) between mature and senescent leaves were Li in *N. pumilio* (LPS site) and Tl in both species and MPS and LPS sites, in all cases concentrations being higher for mature leaves.

### Macronutrient, Micronutrient, and Trace Element Resorption

In general terms and making an average of all sites and species, macronutrient resorption rates showed a decreasing order for N > S = K > P> Mg, being Ca the only element with negative values (i.e., no resorption determined) (**Table 4**). Interactions between sites and species were significant (P < 0.05) for all evaluated elements. Resorption of the majority of the elements did not vary between species in any of the evaluated sites, with the only exception of Mn in the LPS which was higher (60% more) (P < 0.05) for *N. antarctica* leaves. Differences in macronutrient resorption rates between sites were found only for *N. antarctica* leaves, being this parameter lower for trees grown in the most productive site (HPS). In such case, resorption rate decreases of 20% for N, 30% for P, and 20% for S were estimated (**Table 4**). For *N. pumilio* leaf micronutrient and trace element concentrations followed similar trends, with no differences (P > 0.05) in NuR rates recorded between experimental sites. For *N. antarctica* some micronutrients (i.e., Zn) and trace elements (i.e., Sr and Tl) were resorbed at lower rates for the HPS (**Tables 5** and **6**, respectively). On the other hand, regardless of the site or the species, some micronutrients (i.e., Fe) and trace elements (i.e., Al and Ti) were not resorbed.

**Table 4:** Resorption efficiency (%) of macronutrients of *N. pumilio* and *N. antarctica* leaves collected from three sites with contrasting environmental conditions in southern Patagonia.

Species	Sites	Ca	K	Mg	N	P	S
*N. pumilio*	HPS	−37 ± 21a	44 ± 16a	14 ± 20a	63 ± 9a	31 ± 26a	51 ± 8a
	MPS	−16 ± 23a	47 ± 31a	21 ± 18a	62 ± 9a	52 ± 13a	53 ± 7a
	LPS	−18 ± 25a	68 ± 5a	23 ± 21a	63 ± 10a	54 ± 11a	53 ± 11a
*N. antarctica*	HPS	−76 ± 73b	34 ± 32a	17 ± 15a	46 ± 19b	16 ± 18b	36 ± 18b
	MPS	−47 ± 42ab	50 ± 16a	22 ± 24a	66 ± 15a	49 ± 24a	55 ± 11a
	LPS	1 ± 14a	66 ± 20a	25 ± 28a	59 ± 23ab	46 ± 23a	56 ± 19a

For each species, different letters for the same nutrient indicate significant differences (P < 0.05) between sites.

HPS, highly productivity site; MPS, medium productivity site; LPS, low productivity site.

**Table 5:** Resorption efficiency (%) of micronutrients of *N. pumilio* and *N. antarctica* leaves collected from three sites with contrasting environmental conditions in southern Patagonia.

Species	Sites	B	Cu	Fe	Mn	Ni	Zn
*N. pumilio*	HPS	4 ± 31a	4 ± 28a	−26 ± 25a	−21 ± 24a	51 ± 24a	15 ± 23a
	MPS	17 ± 21a	−19 ± 49a	−35 ± 22a	−3 ± 12a	41 ± 39a	32 ± 18a
	LPS	15 ± 7a	10 ± 45a	−178 ± 300a	−56 ± 85a	54 ± 16a	36 ± 16a
*N. Antarctica*	HPS	−17 ± 71a	17 ± 21a	−100 ± 160a	−30 ± 39b	23 ± 44a	1 ± 63b
	MPS	6 ± 19a	−41 ± 58b	−161 ± 305a	−52 ± 70b	35 ± 34a	26 ± 13ab
	LPS	2 ± 46a	30 ± 23a	−31 ± 78a	63 ± 31a	45 ± 64a	42 ± 24a

For each species, different letters for the same nutrient indicate significant differences (P < 0.05) between sites.

HPS: highly productivity site; MPS: medium productivity site; LPS: low productivity site.

**Table 6:** Resorption efficiency (%) of trace elements of *N. pumilio* and *N. antarctica* leaves collected from three contrasting sites in southern Patagonia.

Species	Sites	Al	Li	Pb	Rb	Sr	Ti	Tl
*N. pumilio*	HPS	1 ± 29a	34 ± 31a	10 ± 93a	54 ± 23a	2 ± 17a	−45 ± 31a	56 ± 20a
	MPS	−21 ± 47a	45 ± 12a	−5 ± 63a	54 ± 13a	27 ± 26a	−89 ± 28a	62 ± 8a
	LPS	−157 ± 94a	45 ± 31a	−97 ± 297a	78 ± 13a	−2 ± 48a	−408 ± 666a	48 ± 22a
*N. antarctica*	HPS	−53 ± 141a	−3 ± 43a	47 ± 16a	37 ± 61a	−38 ± 77b	−136 ± 210a	34 ± 14b
	MPS	−123 ± 96a	3 ± 53a	−105 ± 60b	52 ± 38a	−8 ± 49ab	−264 ± 438a	59 ± 19a
	LPS	−4 ± 72a	30 ± 43a	41 ± 21a	42 ± 42a	34 ± 45a	−60 ± 110a	69 ± 4a

For each species, different letters for the same nutrient indicate significant differences (P < 0.05) between sites.

HPS, highly productivity site; MPS, medium productivity site; LPS, low productivity site.

Nutrient resorption was positively correlated (P < 0.05) with mature leaf concentrations for a few micronutrients and trace elements (i.e., Cu, Li, Mn, Ni, and Pb). In the case of soil and mature leaf concentrations, there were only significant (P < 0.05) correlations for N, Fe, and Mg, being the correlation positive for N and Mg and negative for Fe (**Table 7**).

**Table 7:** Pearson correlation coefficient (R) and P value between elements concentration in soil, mature leaves and NuR in leaves of *N. antarctica* and *N. pumilio* from three contrasting sites of southern Patagonia.

Element	Correlation between resorption and mature leaf concentration		Correlation between soil and mature leaf concentration	
	R	p	R	P
N	0.24 (+)	0.21	0.68 (+)	<0.001
C	0.27 (+)	0.16		
Al	0.23 (+)	0.23		
B	0.18 (+)	0.34		
Ca	0.18 (+)	0.36	0.05 (−)	0.781
Cu	0.86 (+)	<0.001		
Fe	0.18 (+)	0.353	0.40 (−)	0.030
K	0.22 (+)	0.255	0.35 (+)	0.059
Li	0.49 (+)	0.008		
Mg	0.36 (+)	0.057	0.62 (+)	<0.001
Mn	0.48 (+)	0.009	0.10 (−)	0.617
Ni	0.44 (+)	0.018		
Pb	0.66 (+)	<0.001		
P	0.33 (+)	0.086	0.01 (−)	0.993
Rb	0.23 (+)	0.249		
S	0.12 (+)	0.542		
Sr	0.09 (+)	0.648		
Ti	0.30 (+)	0.109		
Tl	0.24 (+)	0.265		
Zn	0.01 (+)	0.945		

Signs in parentheses indicate if the correlation is positive (+) or negative (−).

## Discussion

This study focused on analyzing the rate of mineral element resorption in two main *Nothofagus* species of southern Patagonia, considering as hypotheses that: (i) under similar growing conditions *N. pumilio* will be more NuR efficient than *N. antarctica*, and (ii) under harsher climatic conditions both *Nothofagus* species will have increased NuR rates. The overall results gathered for two *Nothofagus* species grown in three study states with different environmental and soil conditions led us to reject both hypotheses (one of them only partially) as discussed in detail in the following paragraphs. When comparing the different quality sites in relation to environmental conditions, it must be for example stressed that during the warmest trimester of the growing season, precipitations were higher for the highest quality location and lower for less productive experimental plots, suggesting a combined positive effect of higher temperature and water availability on tree growth. In general, increased SLA values were recorded for senescent leaves compared with mature ones which may be related to foliar nutrient concentration decreases during senescence.

### Macronutrient Concentrations and Resorption

Generally, for both species grown in the three experimental sites, the macronutrient concentrations of mature leaves were in the range for adequate growth (Epstein and Bloom, 2005). The concentration of macronutrients in *N. pumilio* mature and senescent leaves were similar to those recorded for Tierra del Fuego Island by Frangi et al. (2005). Similarly, the mature leaf macronutrient concentrations determined for *N. antarctica* in this study are within the range described by Peri et al. (2006; 2008) for the same species in southern Patagonia. In this investigation, the environmental condition differences between sites were not found to influence leaf nutrient concentrations of *Nothofagus* species. This is also consistent with the results reported by Frangi et al. (2005) for *N. pumilio* in Tierra del Fuego Island, where mature leaf macronutrient concentrations did not significantly vary between stands grown in an altitudinal gradient. With the exception of soil N concentration which varied between sites, we recorded no major soil mineral element differences that however did not directly reflect on foliar tissue nutrient concentrations. By contrast, Peri et al. (2006; 2008) measured higher macronutrient concentrations in mature leaves of *N. antarctica* stands growing in a better quality site (i.e., 7.8 m of height in mature trees) compared to a lower quality location (5 m of height in mature trees) in southern Patagonia. These results were attributed to soil nutrient availability variations between experimental plots. Nevertheless, Bahamonde et al. (2015) reported similar concentrations in senescent leaves of *N. antarctica* from three contrasting quality sites. It was observed that for several elements better quality sites did not involve higher soil and foliar tissue concentrations. This may be related to the different mechanisms of mineral element acquisition, transport, and storage by plant as reported for various nutrients (Marschner and Rengel, 2012), together with their interaction with climatic variables. For example, working with peach trees Lawrence and Melgar (2018) found that a 5°C air temperature increase delayed leaf senescence such delayed-senescent leaves having lower leaf N and P concentrations. Moreover, when studying the main soil, spatial and climatic variables influencing productivity of mature *N. antarctica* trees in southern Patagonia, Bahamonde et al. (2018a) concluded that temperature and soil texture, but not soil nutrient concentrations, were the most important parameters explaining the final height of mature trees.

The importance of N or P concentrations in forest soils as a limiting factor for tree growth is widely recognized (Aerts and Chapin, 2000; Fisher and Binkley, 2000). The stoichiometry between leaf N and P has been broadly used as an indicator of nutrient growth constraints at a species level (Van den Driessche, 1974). The recommended thresholds reported by Aerts and Chapin (2000) corresponded to foliar N:P ratios higher than 16 as indicative for P limitation, lower than 14 for N limitation and between 14 to 16 as both N and P constraints. In our study, the two evaluated species in all experimental sites had ratios markedly lower than 14, which indicate a generalized N shortage in all trees subjected to investigation. Similarly, Diehl et al. (2008) found N limitations in *N. antarctica* grown in northern Patagonia, and both N and P limitations for *N. pumilio*. Additionally, Fajardo and Piper (2015) reported N limitation in several tree species (including two evergreen *Nothofagus*) of Chilean Patagonia.

Nutrient resorption studies developed with *Nothofagus* species of Patagonia are scarce and have been mainly focused on C, N, P, Ca, and K (Diehl et al., 2003; Diehl et al., 2008; Fajardo and Piper, 2015). The resorption values we obtained for N, P, and K are within the range reported by Diehl et al. (2003) for *N. antarctica* and *N. pumilio* trees grown in northern Patagonia, and Hevia et al. (1999) for N and P resorption by *N. pumilio* in forests central Chile. However, contrary to our preliminary hypothesis, both species were found to have similar resorption efficiency rate for macronutrients. This implies that the described anatomical and physiological differences between these *Nothofagus* species (Peri et al., 2009; Dettmann et al., 2013) do not seem to influence their resorption efficiency under the environmental conditions evaluated in this investigation. Furthermore, this could be related to the fact that restrictive environmental conditions (mainly low temperatures) in southern Patagonia are more important than species-specific differences. Thus, both species growing under the same conditions may experience similar limitations for nutrient absorption and conservation processes. In this study it was observed that leaf mineral element resorption variations in different sites changed between species. While *N. pumilio* did not show differences between sites, for *N. antarctica* macronutrient resorption (excepting K and Mg) was lower in the most productive site. According to the results obtained for both species, our hypothesis had to be partially rejected (not fully for *N. antarctica*), because evidence was gained that the most restrictive conditions for tree growth did not always correlate with the highest NuR efficiency. There are no previous studies comparing NuR rates for *N. pumilio* and *N. antarctica* growing in sites with contrasting environmental conditions. Nevertheless, Frangi et al. (2005) reported similar concentrations of N, K, Mg, and P, in mature and senescent leaves of *N. pumilio* growing at 220 and 540 m a.s.l., in Tierra del Fuego Island which implied similar resorption values between these forests, as also observed in our study. For *N. antarctica*, Peri et al. (2006; 2008) evaluated the nutrient concentration of different organs (i.e., leaves, branches, bark, sapwood, heartwood, and roots) of trees grown at different forest locations of southern Patagonia. These authors reported that the trees growing under better site conditions had higher nutrient concentrations. A higher accumulation of nutrients in woody tissues may help to partially explain the lower resorption rate in the best study site, because stored nutrients in, e.g., stems or roots may act as essential element reservoirs to be mobilized during the next growing season (Jonasson, 1995).

Regarding NuR, Diehl et al. (2003) found a significant positive correlation between N concentration in mature leaves, N resorption and soil N. The authors suggested that under restricted soil N availability in temperate Patagonian forests, plants may reduce the rate of N absorption and leaf accumulation of this element, rather than increasing resorption. In our study, positive correlations were found between soil and mature leaf N and Mg concentrations, but for macronutrients no correlation between mature leaf concentration and resorption was observed.

In the case of Ca, the generally higher concentrations in senescent leaves (i.e., null resorption) are in agreement with previous studies performed with several species and growing conditions, including Patagonian *Nothofagus* (Frangi et al., 2005; Farahat and Linderholm, 2015; Yan et al., 2015). The low foliar Ca resorption rates in these species are likely related to the limited phloem mobility and structural role of this element in cell walls (Fife et al., 2008; Marschner, 2012).

### Micronutrient and Trace Element Concentrations and Resorption

The results of this study represent the first published leaf micronutrient and trace element concentration values of *N. antarctica* and *N. pumilio*. Most of the evaluated micronutrients (e.g., B, Cu, Mn, Ni, and Zn) were in the concentration range considered as sufficient for plant growth (Lambers et al., 2008; Kirkby, 2012). For both species, the only exception was leaf Fe in the lower productivity sites (MPS and LPS), with concentrations below optimal threshold values (Lambers et al., 2008; Kirkby 2012). In the only reported data set of foliar micronutrient and trace element concentrations in this region, Bahamonde et al. (2016b) evaluated grasses growing in *N. antarctica* forests at similar dates during the growing season. Comparing with these grass tissue data, the concentrations measured for *N. antarctica* mature leaves were higher for B, and Zn, and similar for Cu, Mn, and Ni.

Concerning resorption, Mn and Fe showed a general trend to be immobilized in leaves with no resorption regardless of the site or species. However, *N. antarctica* leaves reabsorbed 60% of Mn in the LPS. The very high Mn concentrations measured for mature *N. antarctica* leaves in LPS may be related to the major uptake of this element by *N. antarctica* compared with *N. pumilio*. Furthermore, Mn concentrations in plants have been related to high organic matter content in soils (e.g., Kabata-Pendias, 2011), as we found for the LPS. Killingbeck and Costigan (1988) reported Fe resorption of about 30% in *Quercus* and *Vaccinium* leaves. In our study, there was no relationship between mature leaf concentrations and Fe resorption which may be related to the very low or null mobility of this element in many species (Marschner, 2012).

On the other hand, B, Cu, Ni, and Zn were mostly resorbed. The resorption values for Cu and Ni were positively correlated with concentrations in mature leaves which is consistent with the findings by Killingbeck and Costigan (1988), who observed resorption for Cu and other microelements.

Similarly, the trace elements evaluated (Al, Li, Pb, Rb, Sr, Ti, and Tl) were below the concentration range considered toxic for plants (e.g., Kabata-Pendias, 2011). Compared to the results by Bahamonde et al. (2016b) the concentrations in *N. antarctica* mature leaves evaluated here were higher for Al, Sr, and Ti, lower for Rb, and similar for Pb. It must be noted that some of such trace elements may replace the role of some essential nutrients, like e.g., Rb with K (Nyholm and Tyler, 2000), or Li and Sr with Ca (Shahzad et al., 2016; Sasmaz and Sasmaz, 2017). The results obtained in this study for instance for Rb show a resorption rate similar to that of K which is in agreement with other studies on the mobility of this element (e.g., Nyholm and Tyler, 2000).

Concerning the resorption rate of such trace elements, Al and Ti were immobilized in leaves with no resorption regardless of the site or species. Aluminum and Ti are often found in plant tissues, but their potential physiological and metabolic function are still unclear (Kabata-Pendias, 2011). There is no preliminary information on the potential rate of foliar Al and Ti resorption. We speculate that no resorption of these elements may be related to their low translocation capacity and/or to their high toxicity risk for plants (specially Al) (Kabata-Pendias, 2011; Marschner, 2012). On the other hand, Li, Rb, Pb, and Tl were resorbed. The resorption values for Li were positively correlated with mature leaf concentrations and were similar the results obtained by Killingbeck and Costigan (1988).

### Ecological Implications

Regardless of the physiological and anatomical differences reported between the species analyzed in this investigation (Peri et al., 2009; Dettmann et al., 2013), their macronutrient resorption rates were similar. This may imply that for these species, nutrient conservation may be more related to an intrinsic, species-specific feature rather than to soil fertility. Thus, the co-occurrence of both *N. antarctica* and *N. pumilio* under the same soil and environmental conditions would be given under a scenario where competition for nutrients is not evident. This is consistent with the findings by Diehl et al. (2003), who reported that nutrient performance (i.e., concentrations and resorption rates) differences were marked between life forms (i.e., deciduous broad-leaved, conifers and evergreens), but not between deciduous species in mixed forests (which included *N. antarctica* and *N. pumilio*) of northern Patagonia.

On the other hand, the lower resorption rates in the most productive site of *N. antarctica*, and the lack of differences between sites of *N. pumilio*, could be related to the ecological plasticity of such species. It is widely accepted that *N. antarctica* has the broadest ecological amplitude of south American *Nothofagus* genus (Donoso et al., 2006). Thus, this species is distributed from 36° 30’ to 56° 00` latitude, and from 0 to 2000 m a.s.l. altitude (Veblen et al., 1996). *Nothofagus antarctica* can subsequently grow in contrasting environmental conditions such as poorly drained locations with high precipitations, exposed windy areas with shallow soils or drier sites in the limit with the Patagonian steppe. By contrast, *N. pumilio* thrives under higher soil water availability and its distribution is more restricted to narrower environmental conditions (Veblen et al., 1996). Similarly, Peri et al. (2009) reported that *N. antarctica* had a better response to both water shortage and waterlogging than *N. pumilio*. Hence, the ecological plasticity of these species may partially explain their different rates of NuR in association with environmental conditions changes. This information may be helpful for understanding the potential performance of these species under the current climate change scenario.

According to our results, both *N. antarctica* and *N. pumilio* in southern Patagonia may use trace element resorption as a conservation strategy which may be related to the low availability or restrictions for the acquisition of these elements in soils. Concerning the variations between sites, contrary to N and P which often show significant resorption rates in the majority of the plant species analyzed, the scarce information available on trace element resorption leads to a high result variability, for example, between species grown in the same site, or between years for the same species (see Killingbeck, 2004).

## Conclusions

In this study, we assessed the mineral element resorption rate of two main *Nothofagus* species of Patagonian forest, i.e., *N. antarctica* and *N. pumilio*. Contrary to our preliminary hypotheses, both *Nothofagus* species were found to have a similar nutrient conservation strategy as derived from the lack of NuR differences measured when both species were grown under the same soil and climatic conditions. Furthermore, higher NuR rates were not recorded for the sites with more limiting soil and climatic conditions. Constraints related to plant N availability which seem to widely occur in Patagonian forests, were also identified in this investigation. Our study represents the first published data on the foliar micronutrient and trace element concentrations of both *Nothofagus* spp. grown in Patagonia. Leaf micronutrient concentrations were generally in the sufficiency range reported for plant growth, while trace element concentrations were below the toxicity range. It is concluded that future studies under controlled and field conditions are necessary for improving our understanding of nutrient use efficiency of forest species.

## Data Availability Statement

This study was financed by CONICET (Argentinean National Research Council) project. According to their specifications, at the end of the project and after publishing the results the data can be deposited into an open access repository. Since this belongs to a still ongoing CONICET project, we cannot deposit the data open access repository at this moment.

## Author Contributions

HB contributed to design the study, develop the experiments, analyses and interpret the results, write the draft and approve its final version. VF contributed to design this study, develop the trials, analyses and interpret the results, write, revise and approve the final draft. JG contributed to discussions for the development of the experiments and the interpretation of the results. He also revised the manuscript and approved its final version. FM contributed to samples collection in the field and revised the manuscript. PP contributed to the design the study and revised and approved the final version of the manuscript.

## Funding

This research has been partially financed by Instituto Nacional de Tecnología Agropecuaria (INTA) and Consejo Nacional de Investigaciones Científicas y Técnicas (CONICET), Argentina.

## Conflict of Interest

The authors declare that the research was conducted in the absence of any commercial or financial relationships that could be construed as a potential conflict of interest.

## References

[B1] Álvarez-Fernández A. Pérez-Sanz A. Lucena J. J. (2001). Evaluation of effect of washing procedures on mineral analysis of orange and peach leaves sprayed with seaweed extracts enriched with iron. Commun. Soil Sci Plant Anal. 32 (1-2), 157–170. 10.1081/CSS-100103000

[B2] Aerts R. Chapin F.S. III. (2000). The mineral nutrition of wild plants revisited: are-evaluation of processes and patterns. Adv. Ecol. Res. 30, 1–68. 10.1016/S0065-2504(08)60016-1

[B3] Aerts R. (1996). Nutrient resorption from senescing leaves of perennials: are there general patterns? J. Ecol. 84, 597–608. 10.2307/2261481

[B4] Ares A. Gleason S. M. (2007). “Foliar nutrient resorption in tree species,” in New Research on Forest Ecology. Ed. Scaggs A. K. (New York: Nova Science Publishers), 1–32.

[B5] Bahamonde H. A. Peri P. L. Martínez Pastur G. Monelos L. H. (2015). Litterfall and nutrients return in *Nothofagus antarctica* forests growing in a site quality gradient with different management uses in Southern Patagonia. Eur. J. For. Res. 134 (1), 113–124. 10.1007/s10342-014-0837-z

[B6] Bahamonde H. A. Sosa Lovato S. Gesto E. Martínez Pastur G. Lencinas M. V. Soler R. (2016a). “Variación temporal y espacial de la humedad de suelo y las raíces finas en bosques de ñire bajo uso silvopastoril en la Patagonia sur,” in Book of proceedings of V Jornadas Forestales (Argentina: Esquel), 9–11.

[B7] Bahamonde H. A. Fernández V. Mattenet F. Peri P. L. (2016b). Mineral elements in grasses growing in contrasting environmental conditions in southern Patagonia. New Zeal. J. Agr. Res. 59 (3), 235–249. 10.1080/00288233.2016.1188132

[B8] Bahamonde H. A. Martínez Pastur G. Lencinas M. V. Soler R. Rosas Y. M. Ladd B. (2018a). The relative importance of soil properties and regional climate as drivers of productivity in southern Patagonia’s *Nothofagus antarctica* forests. Ann. For. Sci. 75 (2), 45. 10.1007/s13595-018-0725-7

[B9] Bahamonde H. A. Fernandez V. Gil L. (2018b). Surface properties and permeability to calcium chloride of *Fagus sylvatica* and *Quercus petraea* leaves of different canopy heights. Front. Plant Sci. 9, 494. 10.3389/fpls.2018.00494 29720987 PMC5915543

[B10] Barrera M. D. Frangi J. L. Richter L. L. Perdomo M. H. Pinedo L. B. (2000). Structural and functional changes in *Nothofagus pumilio* forests along an altitudinal gradient in Tierra del Fuego, Argentina. J. Veg. Sci. 11, 179–188. 10.2307/3236797

[B11] Dettmann S. Pérez C. A. Thomas F. M. (2013). Xylem anatomy and calculated hydraulic conductance of four *Nothofagus* species with contrasting distribution in South-Central Chile. Trees 27, 685–696. 10.1007/s00468-012-0824-2

[B12] Diehl P. Mazzarino M. J. Funes F. Fontenla S. Gobbi M. Ferrari J. (2003). Nutrient conservation strategies in native Andean-Patagonian forests. J. Veg. Sci. 14, 63–70. 10.1111/j.1654-1103.2003.tb02128.x

[B13] Diehl P. Mazzarino M. J. Fontenla S. (2008). Plant limiting nutrients in Andean-Patagonian woody species: effects of interannual rainfall variation, soil fertility and mycorrhizal infection. For. Ecol. Manage. 255 (7), 2973–2980. 10.1016/j.foreco.2008.02.003

[B14] Donoso C. Steinke L. Premoli A. (2006). “*Nothofagus antarctica* (G. Forster) Oerst,” in Las especies arbóreas de los bosques templados de Chile y Argentina. Ed. Donoso Zegers C. (Valdivia, Chile: Autoecología), 401–410.

[B15] Epstein E. Bloom A. (2005). Mineral Nutrition of Plants: Principles and Perspectives. 2nd ed Sunderland, Massachusetts: Sinauer Associates, Inc.

[B16] Fajardo A. Piper F. I. (2015). High foliar nutrient concentrations and resorption efficiency in *Embothrium coccineum* (Proteaceae) in southern Chile. Am. J. Bot. 102 (2), 208–216. 10.3732/ajb.1400533 25667073

[B17] Farahat E. Linderholm H. W. (2015). Nutrient resorption efficiency and proficiency in economic wood trees irrigated by treated wastewater in desert planted forests. Agr. Water Manage. 155, 67–75. 10.1016/j.agwat.2015.03.008

[B18] Farr T. G. Rosen P. A. Caro E. Crippen R. Duren R. Hensley S. (2007). The shuttle radar topography mission. Rev. Geophys. 45, RG2004.

[B19] Fife D. N. Nambiar E. K. S. Saur E. (2008). Retranslocation of foliar nutrients in evergreen tree species planted in a Mediterranean environment. Tree Physiol. 28, 187–196. 10.1093/treephys/28.2.187 18055429

[B20] Fisher R. F. Binkley D. (2000). Ecology and Management of Forest Soils. New York: John Wiley and Sons Inc.

[B21] Frangi J. L. Barrera M. D. Puigdefábregas J. Yapura P. F. Arambarri A. M. Richter L. L. (2004). “Ecología de los bosques de Tierra del Fuego,” in Ecología y Manejo de los Bosques de Argentina. Eds. Arturo M. Frangi J. Goya J. F. (La Plata, Argentina: Editorial de la Universidad Nacional de La Plata (EDULP)). Available at: http://sedici.unlp.edu.ar/bitstream/handle/10915/15915/Ecolog%C3%ADa_de_los_bosques_de_Tierra_del_Fuego:Jorge_L._Frangi:Marcelo_D._Barrera:Juan_Puigdef%C3%A1bregas:Pablo_F._Yapura:Ang%C3%A9lica_M._Arrambari_y_Laura_Richter_.pdf?sequence=20 (Accessed ).

[B22] Frangi J. L. Barrera M. D. Richter L. L. Lugo A. E. (2005). Nutrient cycling in *Nothofagus pumilio* forests along an altitudinal gradient in Tierra del Fuego, Argentina. For. Ecol. Manage. 217 (1), 80–94. 10.1016/j.foreco.2005.05.051

[B23] Freschet G. T. Cornelissen J. H. C. van Logtestijn R. S. P. Aerts R. (2010). Substantial nutrient resorption from leaves, stems and roots in a subarctic flora: what is the link with other resource economics traits? New Phytol. 186, 879–889. 10.1111/j.1469-8137.2010.03228.x 20345640

[B24] Hagen-Thorn A. Varnagiryte I. Nihlgård B. Armolaitis K. (2006). Autumn nutrient resorption and losses in four deciduous forest tree species. For. Ecol. Manage. 228 (1-3), 33–39. 10.1016/j.foreco.2006.02.021

[B25] Hevia F. Decker K. L. Boerner R. E. (1999). Foliar nitrogen and phosphorus dynamics of three Chilean *Nothofagus* (Fagaceae) species in relation to leaf lifespan. Am. J. Bot. 86 (3), 447–455. 10.2307/2656765 10077506

[B26] Hijmans R. J. Cameron S. E. Parra J. L. Jones P. G. Jarvis A. (2005). Very high resolution interpolated climate surfaces for global land areas. Int. J. Climatol. 25, 1965–1978. 10.1002/joc.1276

[B27] Ivancich H. Martínez Pastur G. Peri P. L. (2011). Modelos forzados y no forzados para el cálculo de índice de sitio en bosques de *Nothofagus antarctica* en Patagonia Sur. Bosque 32, 135–145. 10.4067/S0717-92002011000200004

[B28] Jonasson S. (1995). Resource allocation in relation to leaf retention time of the wintergreen *Rhododendron lapponicum* . Ecology 76 (2), 475–485. 10.2307/1941206

[B29] Kabata-Pendias A. (2011). Trace elements in soils and plants. 4th ed Boca Raton FL: CRC Press. 10.1201/b10158

[B30] Killingbeck K. T. Costigan S. A. (1988). Element resorption in a guild of understory shrub species: niche differentiation and resorption thresholds. Oikos 53 (3), 366–374. 10.2307/3565537

[B31] Killingbeck K. T. (1986). The terminological jungle revisited: making a case for use of the term resorption. Oikos 46, 263–264. 10.2307/3565477

[B32] Killingbeck K. T. (1996). Nutrients in senesced leaves: key to search for potential resorption and resorption proficiency. Ecology 77, 1716–1727. 10.2307/2265777

[B33] Killingbeck K. T. (2004). “Nutrient resorption,” in Plant cell death processes, 215–226. San Diego, California, USA: Elsevier Academic Press. 10.1016/B978-012520915-1/50017-5

[B34] Kirkby E. (2012). “Introduction, definition and classification of nutrients,” in Marschner’s Mineral Nutrition of Higher Plants, 3rd ed, 3–5. Oxford, UK: Elsevier Academic Press. 10.1016/B978-0-12-384905-2.00001-7

[B35] Lambers H. Chapin S. F. III Pons T. L. (2008). Plant Physiological Ecology. 2nd ed New York: Springer Science and Business Media 623. 10.1007/978-0-387-78341-3

[B36] Lawrence B. T. Melgar J. C. (2018). Variable fall climate influences nutrient resorption and reserve storage in young peach trees. Front. Plant Sci. 9, 1819. 10.3389/fpls.2018.01819 30619397 PMC6304733

[B37] Liu S. Dong Y. Xu L. Kong J. (2014). Effects of foliar applications of nitric oxide and salicylic acid on salt-induced changes in photosynthesis and antioxidative metabolism of cotton seedlings. Plant Growth Regul. 73 (1), 67–78. 10.1007/s10725-013-9868-6

[B38] Lyu S. Wei X. Chen J. Wang C. Wang X. Pan D. (2017). Titanium as a beneficial element for crop production. Front. Plant Sci. 8, 597. 10.3389/fpls.2017.00597 28487709 PMC5404504

[B39] Marschner P. Rengel Z. (2012). “Nutrient availability in soils,” in Marschner’s Mineral Nutrition of Higher Plants. Ed. Marschner P. (San Diego: Academic Press). 10.1016/B978-0-12-384905-2.00012-1

[B40] Marschner P. (2012). Marschner’s Mineral Nutrition of Higher Plants. Ed. Marschner P. (San Diego: Academic Press).

[B41] Mazur R. Sadowska M. Kowalewska Ł. Abratowska A. Kalaji H. M. Mostowska A. (2016). Overlapping toxic effect of long term thallium exposure on white mustard (*Sinapis alba* L.) photosynthetic activity. BMC Plant Biol. 16, 191. 10.1186/s12870-016-0883-4 27590049 PMC5009500

[B42] Nadal M. Schuhmacher M. Domingo J. L. (2004). Levels of PAHs in soil and vegetation samples from Tarragona county, Spain. Environ. Poll. 132, 1–11. 10.1016/j.envpol.2004.04.003 15276268

[B43] Nyholm N. E. I. Tyler G. (2000). Rubidium content of plants, fungi and animals closely reflects potassium and acidity conditions of forest soils. For. Ecol. Manage. 134, 89–96. 10.1016/S0378-1127(99)00247-9

[B44] Pastur G. M. Cellini J. M. Peri P. L. Vukasovic R. F. Fernández M. C. (2000). Timber production of Nothofagus pumilio forests by a shelterwood system in Tierra del Fuego (Argentina). Forest Ecol. Manag. 134(1–3), 153–162. 10.1016/S0378-1127(99)00253-4

[B45] Peri P. L. Gargaglione V. Martínez Pastur G. (2006). Dynamics of above- and below-ground biomass and nutrient accumulation in an age sequence of *Nothofagus antarctica* forest of Southern Patagonia. For. Ecol. Manage. 233, 85–99. 10.1016/j.foreco.2006.06.009

[B46] Peri P. L. Gargaglione V. Martínez Pastur G. (2008). Above- and belowground nutrients storage and biomass accumulation in marginal *Nothofagus antarctica* forests in Southern Patagonia. For. Ecol. Manage. 255 (7), 2502–2511. 10.1016/j.foreco.2008.01.014

[B47] Peri P. L. Martínez Pastur G. Lencinas M. V. (2009). Photosynthetic response to different light intensities and water status of two main *Nothofagus* species of southern Patagonian Forest, Argentina. J. For. Sci. 55, 101–111. 10.17221/66/2008-JFS

[B48] Peri P. L. Lencinas M. V. Bousson J. Lasagno R. Soler R. Bahamonde H. (2016). Biodiversity and ecological long-term plots in Southern Patagonia to support sustainable land management: the case of PEBANPA network. J. Nat. Conserv. 34, 51–64. 10.1016/j.jnc.2016.09.003

[B49] Peri P. L. Monelos L. Díaz B. Mattenet F. Huertas L. Bahamonde H. A. (2019). Estado y uso de los bosques nativos de lenga, siempre verdes y mixtos en Santa Cruz: base para su conservación y manejo. Ed. Revisada CAP (Buenos Aires, Argentina: Ediciones INTA). 108 .pp https://inta.gob.ar/sites/default/files/inta_libro_estado_y_usos_de_los_bosques_de_lenga_siempreverdes_y_mixtos_de_santa_cruz.pdf.

[B50] Pilon-Smits E. A. Quinn C. F. Tapken W. Malagoli M. Schiavon M. (2009). Physiological functions of beneficial elements. Curr. Opin. Plant Biol. 12 (3), 267–274. 10.1007/978-1-4419-9860-6_4 19477676

[B51] Pourrut B. Shahid M. Dumat C. Winterton P. Pinelli E. (2011). Lead uptake, toxicity, and detoxification in plants. In Reviews of Environmental Contamination and Toxicology (New York, NY: Springer) 213, (pp. 113–136). 21541849 10.1007/978-1-4419-9860-6_4

[B52] Pugnaire F. I. Chapin F. S.III (1993). Controls over nutrient resorption from leaves of evergreen Mediterranean species. Ecology 74 (1), 124–129. 10.2307/1939507

[B53] Rasband W. S. (1997–2004). ImageJ. Bethesda, Maryland: National Institutes of Health.

[B54] Sasmaz M. Sasmaz A. (2017). The accumulation of strontium by native plants grown on Gumuskoy mining soils. J. Geochem. Explor. 181, 236–242. 10.1016/j.gexplo.2017.08.001

[B55] Schlesinger W. H. (1991). Biogeochemistry: An Analysis of Global Change. San Diego: Academic Press, Inc.

[B56] Shahzad B. Tanveer M. Hassan W. Shah A. N. Anjum S. A. Cheema S. A. (2016). Lithium toxicity in plants: reasons, mechanisms and remediation possibilities–A review. Plant Physiol. Biochem. 107, 104–115. 10.1016/j.plaphy.2016.05.034 27262404

[B57] Van den Driessche R. (1974). Prediction of mineral nutrient status of trees by foliar analysis. Bot. Rev. 40, 347–394. 10.1007/BF02860066

[B58] Van Heerwaarden L. M. Toet S. Aerts R. (2003). Current measures of nutrient resorption efficiency lead to a substantial underestimation of real resorption efficiency: facts and solutions. Oikos 101, 664–669. 10.1034/j.1600-0706.2003.12351.x

[B59] Veblen T. T. Donoso C. Kitzberger T. Rebertus A. J. (1996). “Ecology of Southern Chilean and Argentinean *Nothofagus* forests,” in The ecology and biogeography of Nothofagus forests. Eds. Veblen T. T. Hill R. S. Read J. (News Haven: Yale University Press), 293–353.

[B60] Venturas M. Fernández V. Nadal P. Guzmán P. Lucena J. J. Gil L. (2014). Root iron uptake efficiency of *Ulmus laevis* and *U. minor* and their distribution in soils of the Iberian Peninsula. Front. Plant Sci. 5, 104. 10.3389/fpls.2014.00104 24723927 PMC3971191

[B61] Vergutz L. Manzoni S. Porporato A. Novais R. F. Jackson R. B. (2012). Global resorption efficiencies and concentrations of carbon and nutrients in leaves of terrestrial plants. Ecol. Monogr. 82 (2), 205–220. 10.1890/11-0416.1

[B62] Vitousek P. M. Howarth R. W. (1991). Nitrogen limitation on land and in the sea: how can it occur? Biogeochem. 13 (2), 87–115. 10.1007/BF00002772

[B63] Wang Z. Fan Z. Zhao Q. Wang M. Ran J. Huang H. (2018). Global data analysis shows that soil nutrient levels dominate foliar nutrient resorption efficiency in herbaceous species. Front. Plant Sci. 9, 1431. 10.3389/fpls.2018.01431 30319680 PMC6168711

[B64] Yan T. Lü X. Yang K. Zhu J. (2015). Leaf nutrient dynamics and nutrient resorption: a comparison between larch plantations and adjacent secondary forests in Northeast China. J. Plant Ecol. 9 (2), 165–173. 10.1093/jpe/rtv034

[B65] Yuan Z. Y. Chen H. Y. (2009). Global-scale patterns of nutrient resorption associated with latitude, temperature and precipitation. Global Ecol. Biogeogr. 18 (1), 11–18. 10.1111/j.1466-8238.2008.00425.x

[B66] Zhao G. Shi P. Wu J. Xiong D. Zong N. Zhang X. (2017). Foliar nutrient resorption patterns of four functional plants along a precipitation gradient on the Tibetan Changtang Plateau. Ecol. Evol. 7 (18), 7201–7212. 10.1002/ece3.3283 28944011 PMC5606856

